# Aberrant local and global neural activation patterns in pediatric Prader–Willi syndrome

**DOI:** 10.3389/fnins.2026.1696114

**Published:** 2026-04-15

**Authors:** Jie Liu, Zhongxin Huang, Jinhua Cai, Min Zhu, Song Peng, Shuang Ding, Longlun Wang, Wei Tang, Chunlan Sun, Jiaxin Su

**Affiliations:** 1 Department of Radiology, Children’s Hospital of Chongqing Medical University, Chongqing, China; 2 Department of Endocrinology, Children’s Hospital of Chongqing Medical University, Chongqing, China; 3 Department of Radiology, Women and Children’s Hospital of Chongqing Medical University, Chongqing, China; 4 Department of Radiology, Chongqing Health Center for Women and Children, Chongqing, China

**Keywords:** functional connectivity, low-frequency fluctuations, magnetic resonance imaging, Prader–Willi syndrome, regional homogeneity

## Abstract

**Purpose:**

Although cognitive disorders in children with Prader–Willi syndrome (PWS) are linked to abnormalities in spontaneous neural activation and functional connectivity (FC), the specific neural activation patterns remain uncertain, especially in young children with PWS.

**Methods:**

The current study set out to explore specific local and global neural activation in pediatric PWS using the amplitude of low-frequency fluctuations (ALFF), regional homogeneity (ReHo), and seed-based whole brain FC. Information was gathered from 35 pediatric PWS patients and 33 healthy controls (HC). Both groups’ ALFF and ReHo values were computed, and FC were constructed on the basis of altered ALFF and ReHo regions. The relationships between altered ALFF, ReHo, and FC and the Griffiths Developmental Scales (GDS) of the PWS group were analyzed using partial correlation analysis.

**Results:**

Both ALFF and ReHo exhibited decreases in occipital lobe, temporal lobe, and cingulate gyrus, and altered ReHo was present in parietal lobe, frontal lobe, and basal ganglia areas. Moreover, ALFF and ReHo also exhibited increases in occipital and temporal lobes. Decreased FC was detected in the visual network (VN), sensorimotor network (SMN), salience network (SAN), and default mode network (DMN). The SMN-, cingulate-, and occipital lobe-related neural activation patterns were significantly positively correlated with the GDS score.

**Conclusion:**

The PWS group was characterized mainly by decreased neuronal physiological function and the ReHo was similar to ALFF but more extensive. The decreased local and global brain neural activation patterns may serve as early physiological indicators of cognitive abnormalities.

## 1 Introduction

Prader–Willi syndrome (PWS) is the first known genetic imprinting disorder caused by loss of paternally expressed imprinted genes on the long arm of chromosome 15 (Prader and Willi, 1956). PWS prevalence is about 1 in 30,000–1 in 10,000 per year worldwide, with unavailable large-scale population-based studies in China (Whittington et al., 2014; Cassidy and Suzanne, 2012). PWS carries a relatively high mortality rate of approximately 3% annually, with a mortality risk six times that of other developmental disorders.

The predominant features of PWS are neonatal hypotonia, progression to developmental delay, early childhood obesity, and mild intellectual disability (Prader and Willi, 1956; Cassidy and Suzanne, 2012). Hyperphagia and obesity are pervasive in PWS, affecting 90–100% of individuals, while cognitive impairment is universal, only a minority of individuals attain a college-level education. PWS performs worse than intellectual disability (ID) peers in action imitation, which may be related to widespread brain dysfunction. Recent clinical studies (Holland et al., 2019; Michel et al., 2016; Salles et al., 2020) on PWS have focused more on adolescent obesity and obsessive-compulsive tendencies in adulthood. However, cognitive impairments in early childhood groups with PWS are crucially understudied clinical phenotypes (Adhikari et al., 2019; Xu et al., 2017).

Blood oxygen level-dependent fMRI (BOLD-fMRI) has been widely used as a non-invasive functional neuroimaging technique (Lan et al., 2021; Qian et al., 2018), comprising two main methodologies: task fMRI (t-fMRI) and resting-state fMRI (rs-fMRI). Compared with t-fMRI, rs-fMRI is more suitable for young children with poor adherence. In rs-fMRI, brain regions exhibiting similar activity are considered to constitute a coordinated functional brain network. The research techniques for rs-fMRI include two categories: the first is to directly observe spontaneous neuronal activity, such as low-frequency amplitude (ALFF) at baseline brain state, and the other is to evaluate neuronal activity by indirectly observing the association of local neurons with other brain regions, including local coherence (ReHo), seed-based functional connectivity (seed-based FC), etc. Multiparameter functional techniques, which encompass both direct and indirect measurements, provide valuable indications regarding the neurobiological underpinnings of developmental and maturational conditions (Huang and Cai, 2023).

Functional neuroimaging studies have shown that developmental (Vogels et al., 2003) and psychiatric conceptions of cognitive and behavioral phenotypes (Kleinhans et al., 2008) in children with PWS are associated with alterations in brain networks (Huang and Cai, 2023). For example, reduced intranetwork functional connectivity (FC) among the four neural networks (dorsal attention network, the auditory network, the medial visual network and the sensorimotor network) in PWS patients aged 2–6 years was positively correlated with developmental quotients in PWS children (Zhang et al., 2013). The functional network in the right parahippocampal gyrus and the right pallidum showed a positive correlation with developmental scales of the PWS children aged 1.5–6 years (Huang et al., 2024).

Several functional studies have identified brain regions (the prefrontal, right inferior frontal, anterior cingulate cortices, limbic structure, and basal ganglia) of PWS aged 6–45 years that are relevant to behavioral and developmental features, such as autism spectrum disorder, food-related functioning, obsessive behavior, and task-switching difficulty(Yamada et al., 2021; Pujol et al., 2016). The hyposensitivity of GABA receptors is likely the cause of the corticospinal hypoexcitability in PWS.

However, most of the aforementioned studies have not included PWS children, with only two focusing on children aged 6 or younger, and one of them utilised a single activation pattern to assess functional status, thereby impeding the broader significance of these findings.

The present study was aimed at utilizing these advanced neuroimaging techniques to investigate spontaneous neuronal activity alterations associated with early developmental delays in pediatric patients with PWS. We hypothesized that the PWS group would exhibit the following characteristics: (1) ALFF and ReHo; (2) lower whole-brain FCs constructed based on regions with altered ALFF and ReHo; and (3) altered local and global neural activity indicators that were negatively correlated with developmental scale scores. This study was aimed at understanding the potential neuropathological mechanisms of cognitive function and developmental delays in pediatric PWS patients, as well as screening for neuroimaging-related activation patterns that could facilitate the early diagnosis of PWS and the assessment of developmental delays.

## 2 Research design and methods

### 2.1 Subjects

Ethical approval for the study was provided by our institution’s Human Research Ethics Committee, and we obtained written consent from all parents. The registration number is ChiCTR2100046551.

Inclusion criteria for patients with PWS: (1) genetically diagnosed PWS (Cassidy and Suzanne, 2012); and (2) aged 3 months to 6 years. Exclusion criteria for patients with PWS: (1) other mental disorders history. For example, Mental disorders not associated with PWS: schizophrenia spectrum disorders, bipolar disorder, generalised anxiety disorder; conditions that may coexist with PWS but require differential diagnosis: autism spectrum disorders, obsessive-compulsive disorder; (2) a history of psychotropic medication; and (3) any contraindication to magnetic resonance imaging (MRI).

Inclusion criteria for HC: (1) age- and sex-matched; (2) normal intelligence and neurological assessments; and (3) typically developing children who required MRI scans for objective reasons. Exclusion criteria for the HC group are congruent with the PWS group.

The T1-weighted anatomic images were checked by two experienced radiologists to exclude visible lesions. Following MRI scans and data processing, one child of the PWS group with excessive head movement was excluded (translation > 2.0 mm or rotation > 2°). The study included 68 subjects: 35 pediatric patients with PWS (11 females; mean age ± SD: 3.39 ± 3.38 years) and 33 HC (14 females; mean age ± SD: 4.22 ± 1.75 years). All participants underwent classical laboratory tests, a series of neuropsychological and neurodevelopmental tests. The details of neuropsychological and neurodevelopmental tests are provided in the Supplementary materials.

### 2.2 Neuropsychological and neurodevelopmental tests

All participants completed a series of neuropsychological and neurodevelopmental tests targeting the relevant cognitive areas. After a battery of neuropsychological and neurodevelopmental tests administered by experienced pediatricians, such as the Gesell Developmental Schedule and the Stanford Binet Intelligence Scale (SBIS), typically developing children with reports of normal growth, normal intelligence, and normal neurological examinations were recruited and defined as healthy controls (HCs). The Griffiths Development Scales (GDS) were used to assess all PWS patients and HCs.

The Gesell Development Schedule is a classic development assessment scale that has been widely used to assess neurodevelopmental statuses in children aged 0 through 6 years. This scale contains five separate subscales as follows: gross motor, fine motor, adaptive behavior, language, and personal–social behavior (Cassidy and Suzanne, 2012; Holland et al., 2019; Michel et al., 2016). The developmental quotient is classified into three types, namely, normal (≥ 85), borderline (76–84), or delayed (≤ 75).

The cognitive ability of the HCs was assessed using the Stanford Binet Intelligence Scale (SBIS). This assessment was designed for individuals spanning the ages of 2–85 years. The composite score and each tested scale/factor showed good reliability (composite score *α* = 0.98; nonverbal *α* = 0.95; verbal *α* = 0.96; factors *α* = 0.88–0.91).

The GDS were used to assess the mental development of all participants aged 0 through 6 years. Before conducted the assessment, it was ensured that all participants were well rested and in a suitable condition to undergo testing. The GDS provide a general developmental quotient (GQ) with subscales assessing the following skill areas: locomotor (subscale A), personal-social (subscale B), hearing and speech (subscale C), eye-hand coordination (subscale D) and performance (subscale E). A developmental delay is considered present when the subscale quotient or GQ is below the mean (subscale quotient or GQ < 70) by at least 2 standard deviations.

### 2.3 MRI

The experiments were performed on a 3.0 Tesla MRI scanner (Philips Achieva). Sleep deprivation was performed via intravenous propofol (loading dose of 1 mg/kg, followed by 200–300 μg/kg/min) in the PWS and HC groups in order to keep the head still during the scan and improve the quality of the neurological image.

A three-dimensional turbo field echo (TFE) sequence was used to acquire T1-weighted anatomical images. The parameters were as follows: repetition time (TR) = 7.4 ms, echo time (TE) = 3.8 ms, slice thickness = 1 mm, gap = 0 mm, number of slices = 260, field of view (FOV) = 250 mm × 250 mm, flip angle = 8°, acquisition matrix = 228 × 227, voxel size = 0.60 mm × 1.04 mm × 1.04 mm, and total time = 4 min 16 s.

Resting-state fMRI images were collected using an echo planar imaging (EPI) sequence with the following parameters: TR = 2000 ms, TE = 35 ms, flip angle = 90°, 33 axial slices, slice thickness = 3.75 mm, FOV = 240 mm × 240 mm, acquisition matrix = 80 × 78, and voxel size = 3.75 mm × 3.75 mm × 3.75 mm with no gap. One functional run was acquired, and the fMRI scanning session lasted for 8 min 06 s.

Image preprocessing was carried out in MATLAB 2022b (MathWorks Inc., Natick, MA, USA) using the Data Processing Assistant for Resting-State fMRI (DPARSF; http://rfmri.org/DPARSF) through statistical parametric mapping (SPM12; http://www.fil.ion.ucl.ac.uk/spm/) and the Resting-State fMRI Data Analysis Toolkit plus V1.32 (RESTplus V1.32; http://restfmri.net/forum/restplus) software.

First, convert DICOM to NIFTI format and discard the first 10 volumes. Next, perform slice timing correction and realignment for head motion correction. Then, coregister the high-resolution T1-weighted images. Finally, resample the images to 3-mm isotropic voxels and normalize them to the Montreal Neurological Institute (MNI) template.

### 2.4 ALFF and ReHo analyses

ALFF and ReHo analyses were performed employing RESTplus software (Kiviniemi et al., 2004; Zang et al., 2004).

For the ALFF analysis, the resampled images were smoothed (6-mm Gaussian kernel) to reduce the degree of bad normalization. Apply linear trending and bandpass filtering (0.01–0.08 Hz) to minimize the impact of low-frequency drift. The nuisance covariates included head motion parameters in 24 directions as well as four mean intermixed signals (from the cerebrospinal fluid, white matter, gray matter, and the whole brain), which were regressed to minimize the effects of nonneuronal signals.

Conduct a ReHo analysis on the preprocessed images. After the linear trend stage, 4 covariates (Friston 24, a global brain signal, a white matter signal, and a cerebrospinal fluid signal) were removed, and bandpass filtering was performed.

### 2.5 Selection of the region of interest (ROI)

After a correction for multiple comparisons was performed using false discovery rate (FDR) correction, the brain regions with ALFF and ReHo values (FDR, *p* < 0.05, clusters >15 voxels) in the PWS group were extracted as ROIs, and small spherical ROIs with radii of 5 mm were formed.

### 2.6 Data statistical analysis

Two-sample *t* tests were used to compare the ALFF values and ReHo values between the two groups via the RESTplus toolbox.^1^ Age, sex, and propofol dosage, as covariates, were removed via regression to reduce the effect of confounding. The false discovery rate (FDR) method (*p* < 0.05) was used to correct the comparison results.

The average time series of the ROIs were extracted, and voxel-based functional connections were made with the whole brain. After controlling for age, sex, and propofol dosage, for seed-based whole-brain FC comparisons, statistical correction was performed via a two-sample *t*-test (FDR-corrected *p* < 0.05, cluster threshold >15 voxels).

The ALFF, ReHo, and FC values of PWS group differences were extracted. Correlations were determined and quantified between ALFF, ReHo, and FC values and developmental scales. This was achieved by utilising SPSS software. After controlling for age and sex, partial correlation analysis was performed using a two-tailed test, with statistical significance set at *p* < 0.05.

## 3 Results

### 3.1 Demographic, laboratory and cognitive characteristics

The intergroup participant profiles and biochemical results comparisons are presented in Table 1. The body mass index, total serum cholesterol, and insulin-like growth factor-binding protein 3 values were significantly different between the PWS group and the control group (*p* = 0.002; *p* = 0.011; *p* < 0.001, respectively). The intergroup differences concerning the following parameters were not significant: age, fasting blood glucose, triglycerides, low-density lipoprotein, high-density lipoprotein, insulin, and insulin-like growth factor 1 (*p* > 0.05). The GDS data are shown in Table 2. Developmental delays were present in all domains of the PWS group, among which the practical reasoning quotient was the most severe (58.3%).

**Table 1:** Demographic, clinical, and developmental scales of participants included in the study.

Characteristics	PWS patients (*n* = 35)	Control subjects (*n* = 33)	*p* value
Age (months)	33.26 ± 16.97	34.88 ± 16.51	0.729
Man *n* (%)	23 (66%)	21 (64%)	0.994
Height (cm)	93.39 ± 4.24	96.47 ± 12.33	0.336
Weight (kg)	17.69 ± 1.41	15.73 ± 4.07	0.225
BMI (kg/m^2^)	19.10 ± 0.22	16.68 ± 1.04	0.002^*^
Fasting blood glucose (mmol/L)	5.15 ± 0.08	5.18 ± 0.01	0.973
Serum total cholesterol (mmol/L)	4.16 ± 0.04	4.37 ± 0.61	0.011^*^
Triglycerides (mmol/L)	1.31 ± 0.13	1.28 ± 1.18	0.454
high-density lipoprotein (mmol/L)	1.25 ± 0.13	1.42 ± 0.16	0.762
Low-density lipoprotein (mmol/L)	2.42 ± 0.10	2.60 ± 0.41	0.086
Insulin (μIU/mL)	7.21 ± 4.39	6.63 ± 5.94	0.585
IFG-1 (mg/Ml)	141.89 ± 8.49	137.79 ± 37.73	0.451
IGF BP-3 (μg/mL)	4.06 ± 0.56	2.62 ± 1.80	<0.001^*^

Data are presented as mean ± standard. BMI, body mass index; PWS, Prader-Willi syndrome. ^*^, significant difference.

**Table 2:** Assessment of the Griffith Developmental Scale in children with PWS.

Griffith scale (subscales A–F)	*n* = 35	Retardation^a^ *n* (%)
General quotient (GQ)	62.02 ± 10.20	34 (97.1%)
Locomotor quotient (AQ)	56.51 ± 26.73	32 (91.4%)
Personal-social quotient (BQ)	67.46 ± 1.27	25 (71.4%)
Hearing and language quotient (CQ)	62.76 ± 2.97	31 (88.6%)
Eye-hand coordination quotient (DQ)	67.9 ± 6.08	29 (82.9%)
Performance quotient (EQ)	63.01 ± 14.34	28 (80.0%)
Practical reasoning quotient (FQ)^b^	77.35 ± 4.67	7/12 (58.3%)

Data are presented as mean ± standard or deviation or *n* (%).

a A, GQ, or a subscale quotient <70.

^b^FQ was measured for n children (2–6 year olds) in the sample. PWS, Prader–Willi syndrome.

### 3.2 Statistical imaging results

#### 3.2.1 Analysis of the between-group ALFF values

**Figure 1:**
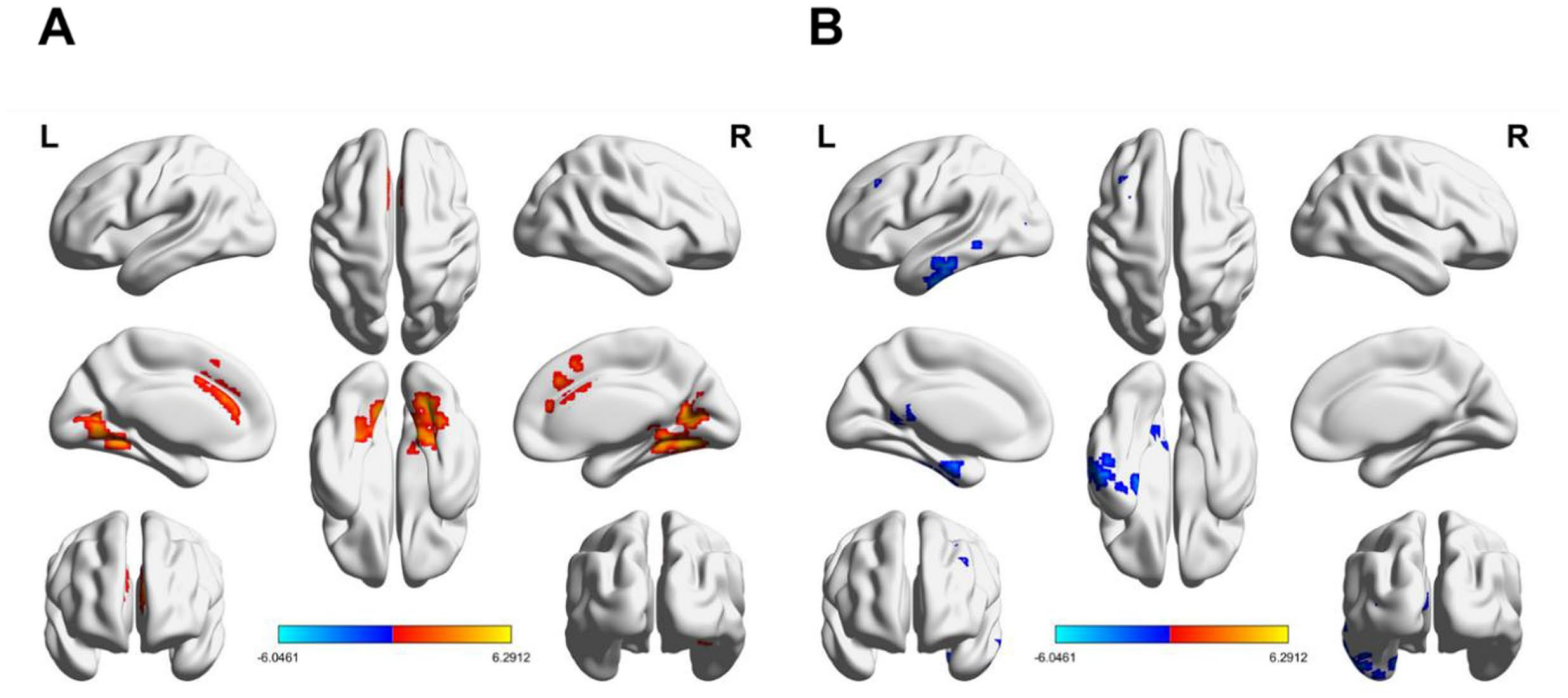
Differences in ALFF values between the PWS group and HCs (*p* < 0.05, FalseDiscovery Rate corrected, cluster >20 voxels). Red/blue regions represent the ALFF values of HCs as greater/lower than the PWS group. ALFF, amplitude of low-frequency fluctuation; PWS, Prader–Willi syndrome; HCs, healthy controls.

Compared with those in the control group, the ALFF values of PWS were significantly increased in temporal lobe [inferior temporal gyrus (ITG. L)], occipital lobe [left middle occipital gyrus (MOG. L)], and left precuneus (PCUN. L), whereas the ALFF values were lower in occipital lobe (bilateral calcarine), temporal lobe [right fusiform gyrus (FFG. R)], and left median cingulate and paracingulate gyri (DCG. L) (*p* < 0.05, FDR-corrected) (see details in Supplementary Table 1, Figure 1).

#### 3.2.2 Analysis of the between-group ReHo values

The brain regions with altered ReHo values were more extensive than those with altered ALFF values. Compared with those in the controls, the increased ReHo brain regions in PWS group were scattered throughout brain, including temporal lobe [bilateral inferior temporal gyrus and the left middle temporal gyrus (MTG. L)] and occipital lobe [left posterior cingulate gyrus (PCG. L) and MOG. L].

**Figure 2:**
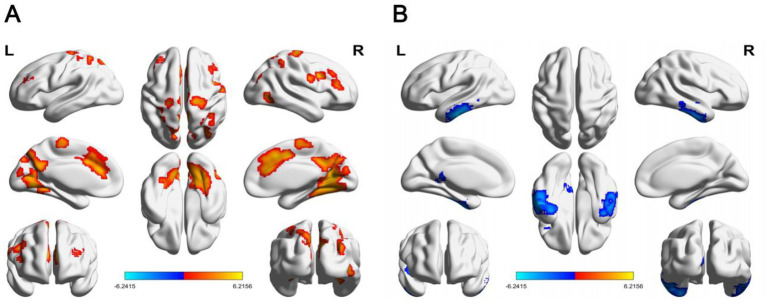
Differences in ReHo values between PWS group and HCs (*p* < 0.05, FalseDiscovery Rate corrected, cluster >50 voxels). Red/blue regions represent the ReHo values of HCs as greater/lower than the PWS group. ReHo, regional homogeneity; PWS, Prader–Willi syndrome; HCs, healthy controls.

In PWS group, decreased ReHo values were more concentrated and symmetrical, and they were primarily centered in bilateral temporal lobe, occipital lobe, parietal lobe, frontal lobe and basal ganglia area, covering the following 5 brain regions: (1) temporal lobe, including the inferior temporal gyrus; (2) occipital lobe, including the bilateral calcarine and right middle occipital gyrus (MOG. R); (3) parietal lobe, including the bilateral postcentral gyrus and left superior parietal gyrus (SPG. L); (4) frontal lobe, including right inferior frontal gyrus, left middle frontal gyrus (MFG. L), right precentral gyrus (PreCG. R) and left paracentral lobule (PCL. L); and 5 basal ganglia areas, including the right caudate nucleus (CAU. R), left lenticular nucleus, putamen (PUT. L), and left median cingulate and paracingulate gyri (*p* < 0.05, FDR-corrected) (see details in Supplementary Table 2, Figure 2).

#### 3.2.3 Seed-based FC results

Five brain regions were chosen as ROIs based on the findings from the between-group ALFF and ReHo value analyses (Supplementary Tables 1, 2). The five brain regions were as follows: ROI 1 in the left calcarine (CAL. L), which belongs to the visual network (VN) (Long et al., 2019); ROI 2 in the right calcarine (CAL. R), which belongs to the VN; ROI 3 in the left median cingulate and paracingulate gyri, which belongs to the salience network (SAN); ROI 4 in the left paracentral lobule, which belongs to the sensorimotor network (SMN); and ROI 5 in left postcentral (PoCG. L), which belongs to the SMN.

**Figure 3:**
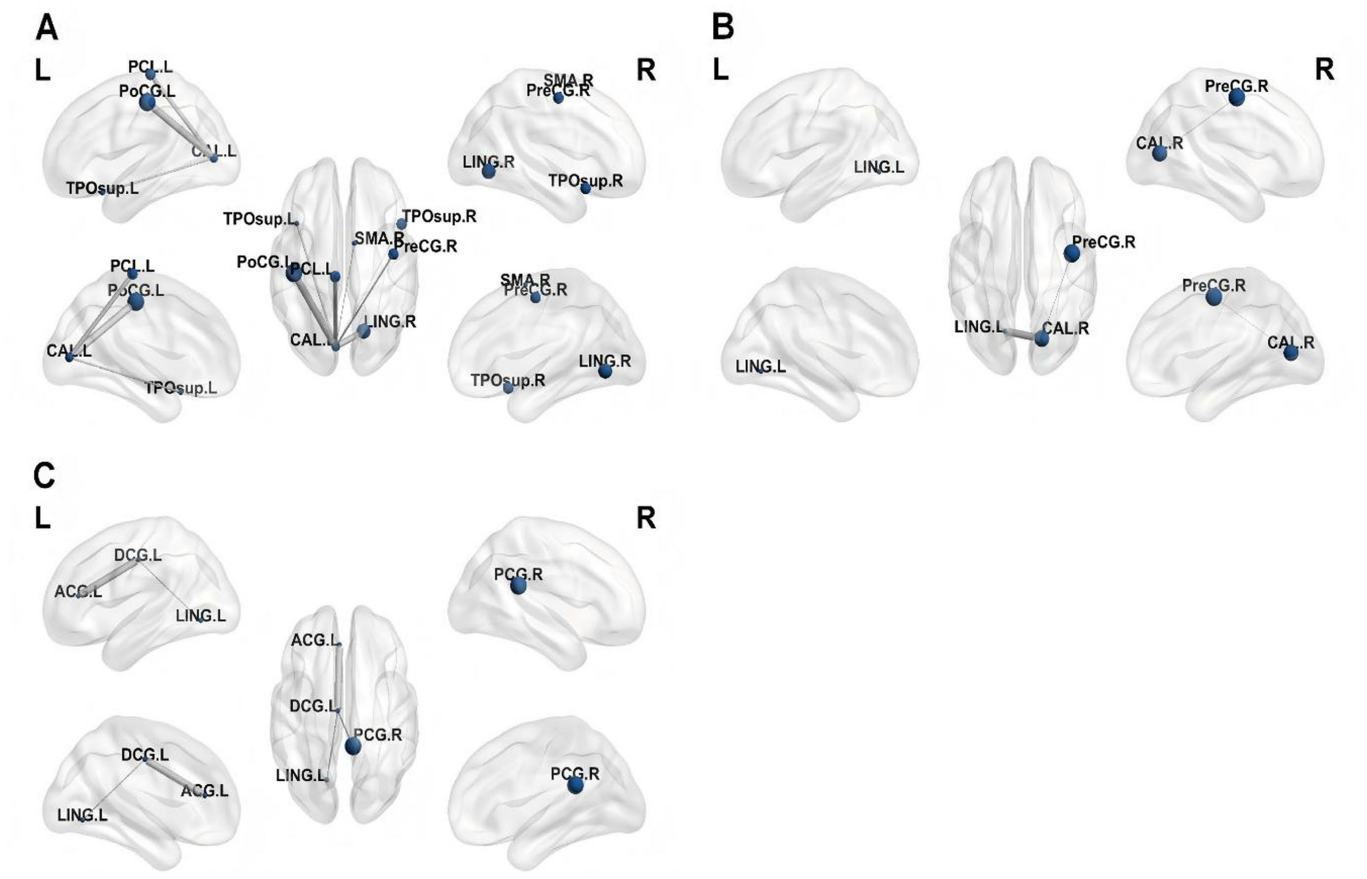
Utilizing brain regions with different ALFF values as seeds, including bilateral CAL, DCG. L, it was discovered that they exhibited decreased functional connectivity with other voxels of the whole brain (*p* < 0.05, FalseDiscovery Rate corrected). CAL, calcarine; DCG. L, left median cingulate and paracingulate gyri.

**Figure 4:**
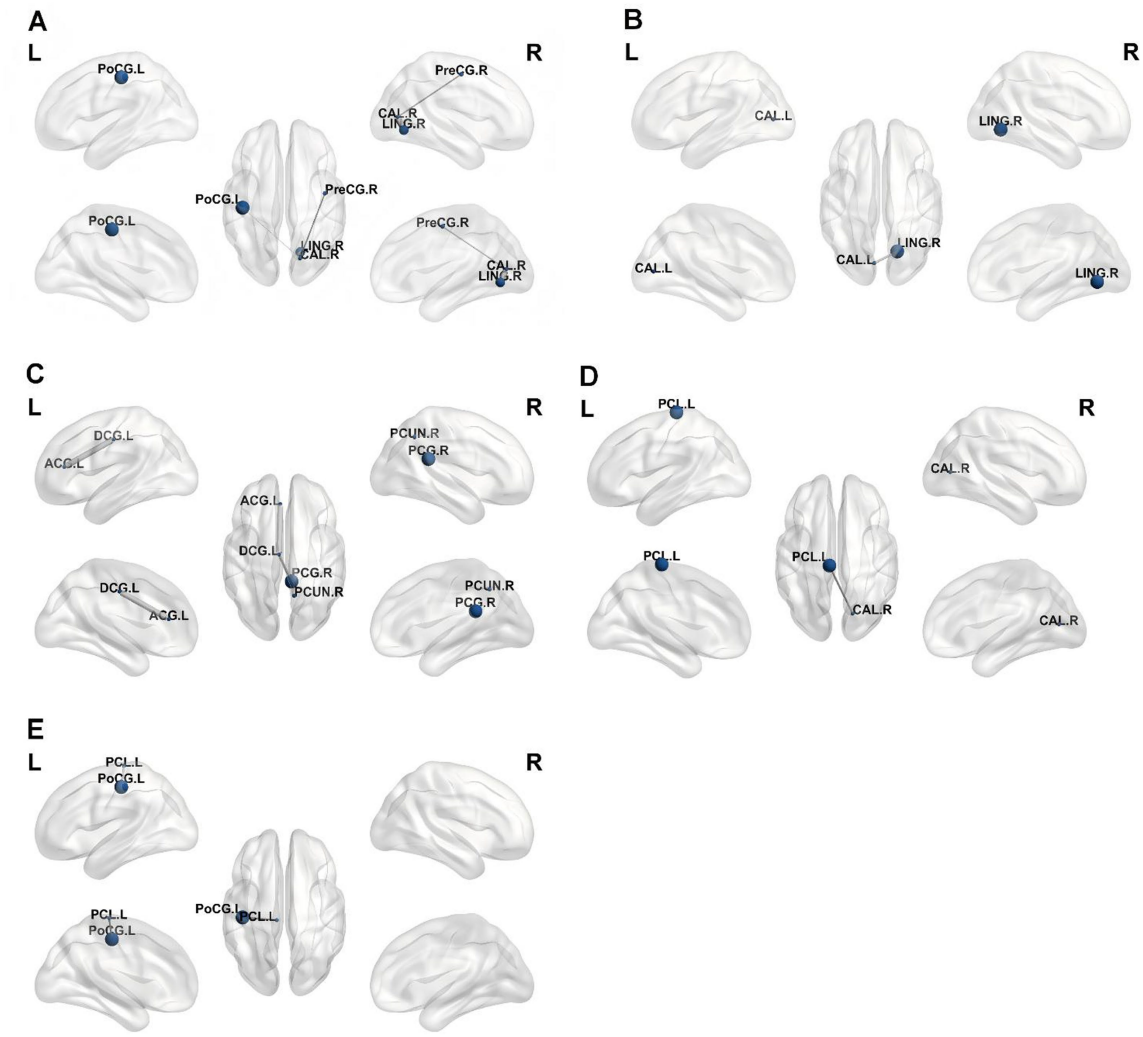
Utilizing brain regions with different ReHo values as seeds, including the bilateral CAL, DCG. L, PCL. L, and PoCG. L, it was discovered that they exhibited decreased functional connectivity with other voxels of the whole brain (*p* < 0.05, False Discovery Rate corrected). CAL, calcarine; DCG. L, left median cingulate and paracingulate gyri; PCL. L, left paracentral lobule; PoCG. L, left postcentral.

The PWS group, including VN [right lingual gyrus (LING. R)] and SMN [left postcentral, right precentral gyrus, left paracentral lobule, and right supplementary motor area (SMA. R)] exhibited decreased FC with ROI 1 (Supplementary Tables 3, 4, Figures 3A, 4B). The PWS group, including the VN (bilateral lingual) and the SMN (right precentral gyrus and left postcentral), exhibited decreased FC with RO1 2 (Supplementary Tables 3, 4, Figures 3B, 4A).

The PWS group, including the DMN [anterior cingulate and paracingulate gyri (ACG. L), right posterior cingulate gyrus (PCG. R), and left precuneus] and VN [left lingual gyrus (LING. L)] exhibited decreased FC with ROI 3 (Supplementary Tables 3, 4, Figures 3C, 4C). PWS was associated with decreased FC between ROI 4 and right calcarine (Supplementary Table 4, Figure 4D), and PWS was linked to decreased FC between ROI 5 and the left paracentral lobule (*p* < 0.05, FDR-corrected) (Supplementary Table 4, Figure 4E).

### 3.3 Correlation analysis

**Figure 5:**
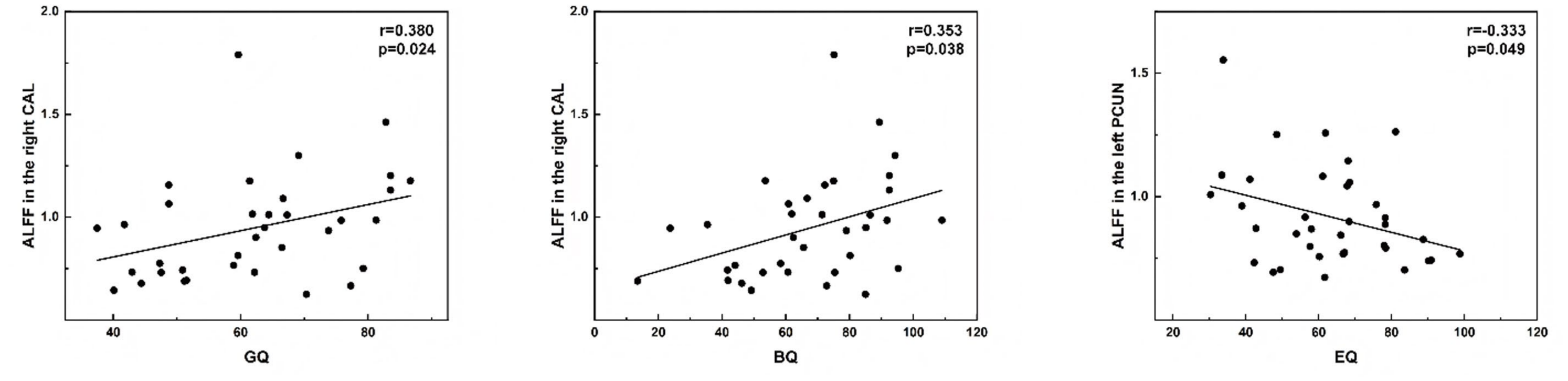
Correlations between ALFF values and GDS in PWS group (*p* < 0.05, two-tailed partial correlation analysis). ALFF, Amplitude of low-frequency fluctuation; GDS, Griffiths Developmental Scales; PWS, Prader–Willi syndrome; HCs, healthy controls; CAL, calcarine; PCUN, precuneus; GQ, general quotient; BQ, personal-social quotient; EQ, performance quotient.

**Figure 6:**
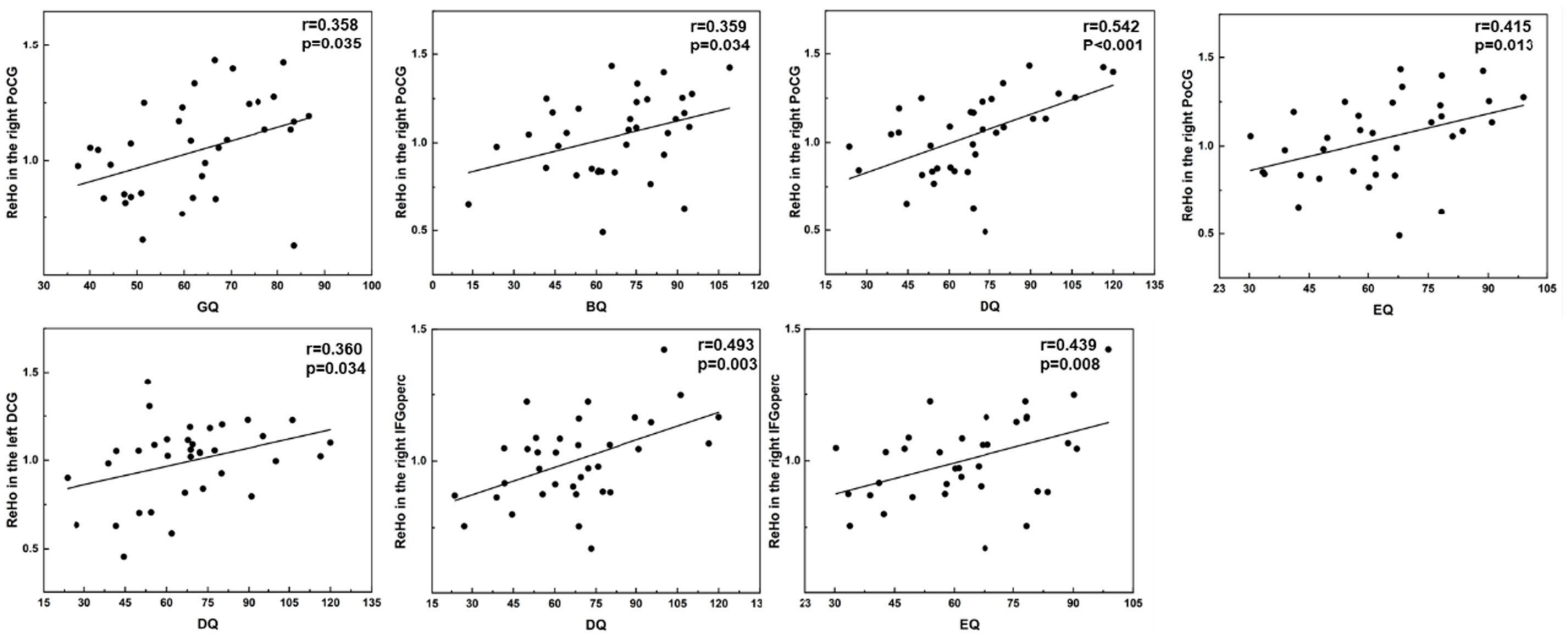
Correlations between ReHo values and GDS in PWS group (*p* < 0.05, two-tailed partial correlation analysis). ReHo, regional homogeneity; GDS, Griffiths Developmental Scales; PWS, Prader–Willi syndrome; HCs, healthy controls; PoCG, postcentral gyrus; DCG, median cingulate and paracingulate gyri; IFGoperc, inferior frontal gyrus, opercular part; GQ, general quotient; BQ, personal-social quotient; DQ, eye-hand coordination quotient; EQ, performance quotient.

**Figure 7:**
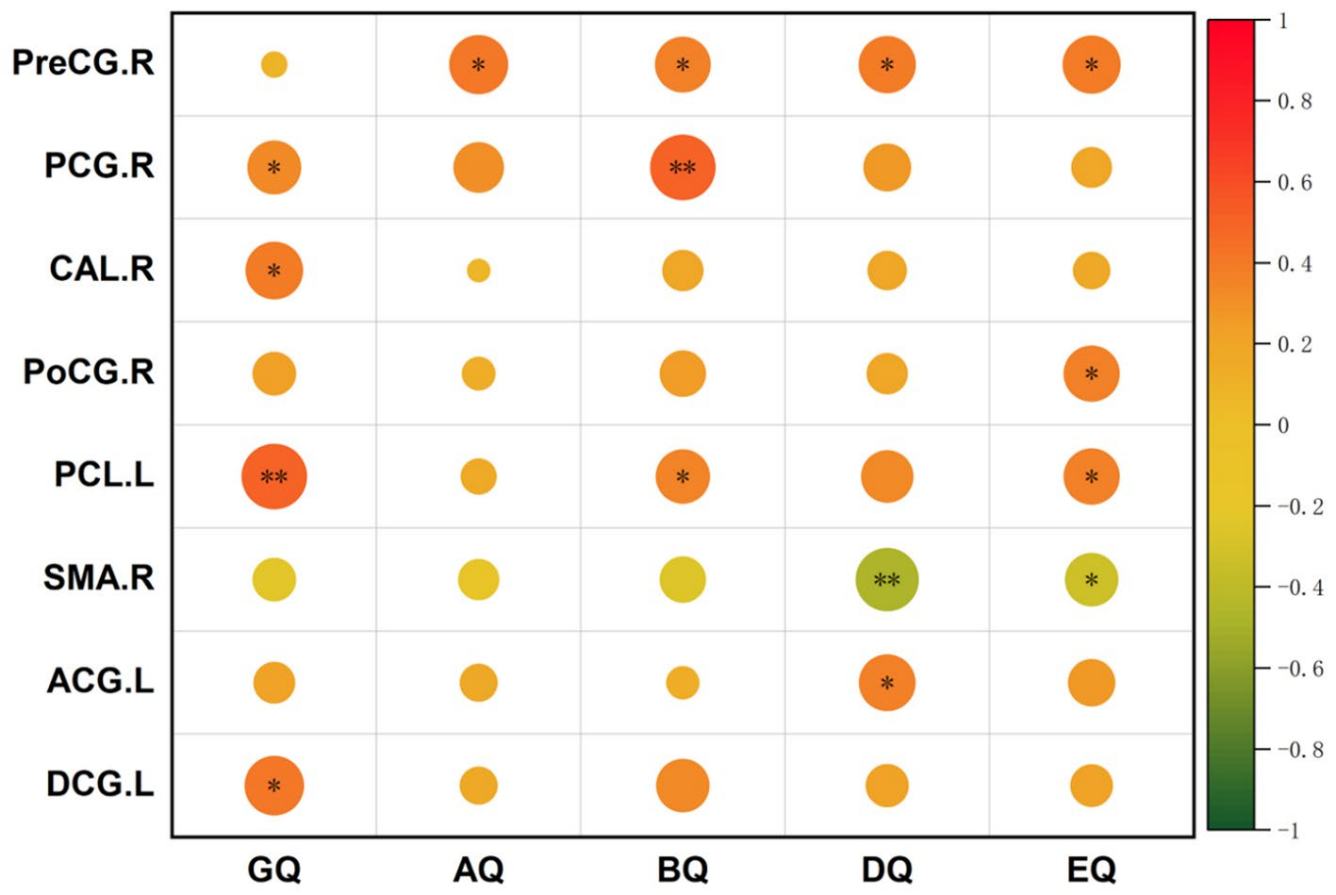
Correlations between FC values and GDS in PWS group (*p* < 0.05, two-tailed partial correlation analysis). FC, functional connectivity; GDS, Griffiths Developmental Scales; PWS, Prader–Willi syndrome; PreCG, precental gyrus; PCG, posterior cingulate gyrus; CAL, calcarine; PoCG, postcentral gyrus; PCL, paracentral lobule; SMA, supplementary motor area; ACG, anterior cingulate and paracingulate gyri; DCG, median cingulate and paracingulate gyri; GQ, general quotient; AQ, locomotor quotient; BQ, personal-social quotient; DQ, eye-hand coordination quotient; EQ, performance quotient.

The PWS brain regions observed based on ALFF, ReHo, and FC analyses that showed a significant positive correlation with the GDS were mainly in the SMN [right postcentral (PoCG. R), right precentral gyrus, and left paracentral lobule], cingulate (left median cingulate and paracingulate gyri, right posterior cingulate gyrus, and left anterior cingulate and paracingulate gyri), and occipital lobe (bilateral calcarine) (Figures 5–7).

The ALFF value in the right calcarine was significantly positively correlated with GQ (*r* = 0.353, *p* = 0.038). Additionally, the ALFF value in the right calcarine was positively correlated with the personal–social quotient (BQ) (*r* = 0.380, *p* = 0.024). Conversely, the ALFF value in the left precuneus exhibited a significant negative correlation with the performance quotient (EQ) in the PWS group (*r* = −0.333, *p* = 0.049) (Figure 5).

The ReHo value in the right postcentral gyrus was positively correlated with the GQ, BQ, eye–hand coordination quotient (DQ), and EQ (*r* = 0.358, *p* = 0.035; *r* = 0.359, *p* = 0.034; *r* = 0.542, *p* < 0.001; and *r* = 0.415, *p* = 0.013, respectively). The ReHo values in the left paracingulate gyri and inferior frontal gyrus, opercular part (IFGoperc. R) were positively correlated with DQ (*r* = 0.360, *p* = 0.034; *r* = 0.493, *p* = 0.003, respectively). The ReHo value in the IFGoperc. R was positively correlated with the EQ score (*r* = 0.439, *p* = 0.008) in the PWS group (Figure 6).

The FC value in the right precentral gyrus was positively correlated with the locomotor quotient (AQ), BQ, DQ, and EQ (*r* = 0.411, *p* = 0.014; *r* = 0.363, *p* = 0.032; *r* = 0.383, *p* = 0.023; and *r* = 0.391, *p* = 0.020, respectively). The FC value in the right posterior cingulate gyrus was positively correlated with the GQ and BQ (*r* = 0.336, *p* = 0.048; *r* = 0.504, *p* = 0.002, respectively). The FC value in the right calcarine was positively correlated with the GQ (*r* = 0.386, *p* = 0.022). The FC value in the right postcentral was positively correlated with the EQ (*r* = 0.366, *p* = 0.031). The FC value in the left paracentral lobule was positively correlated with the GQ, BQ, and EQ (*r* = 0.501, *p* = 0.002; *r* = 0.348, *p* = 0.041; and *r* = 0.371, *p* = 0.028, respectively). The FC value in the anterior cingulate and paracingulate gyri was positively correlated with the DQ (*r* = 0.372, *p* = 0.028). The FC value in the left paracingulate gyri was positively correlated with the GQ (*r* = 0.415, *p* = 0.013), whereas the FC value in the SMA. R was negatively correlated with the DQ and EQ in the PWS group (*r* = −0.464, *p* = 0.005; *r* = −0.335, *p* = 0.049, respectively) (Figure 7).

## 4 Discussion

The present study revealed distinctive neuronal physiological attributes and brain alterations in the functional domains of early childhood patients with PWS, revealing 3 key findings. First, the PWS group was characterized mainly by decreased local neural activation patterns. The brain regions with altered ReHo were similar to those with altered ALFF but more extensive. Both indices suggested decreased activity in occipital lobe, temporal lobe, and cingulate gyrus, but altered ReHo was also present in parietal lobe, frontal lobe, and basal ganglia area. Second, intriguing global neural activation patterns emerged, revealing decreased FCs between the VN and SMN, between the VN and SAN, between the SAN and DMN, and between the SMN and VN in the PWS group. Finally, there was a significant positive correlation between PWS brain regions and GDS, including SMN (right postcentral, right precentral gyrus, and left paracentral lobule), cingulate (left median cingulate and paracingulate gyri, right posterior cingulate gyrus, and left anterior cingulate and paracingulate gyri), and occipital lobe (bilateral calcarine). These findings suggested the presence of discernible local and global neural activation differences between the brains of early childhood children with PWS and those of matched healthy controls. The hyposensitivity of GABA receptors is likely the cause of the reduced local and global neural activation in PWS. More importantly, these results indicated that brain neuronal physiological attributes and functional alterations are linked to clinical symptoms. These clinical symptoms may be caused by cortical dysplasia and a defect in the gamma-aminobutyric acid (GABA)-type A receptor.

### 4.1 Altered local neural activation patterns in PWS patients

Although the ReHo alterations showed a more extensive distribution than the ALFF alterations did in PWS patients, they presented some similarities.

As physiological indicators, both ReHo and ALFF reflect spontaneous local neural activity. A strong positive link between the two indices has been reported in previous studies (Yuan et al., 2013), and strong correlations with cerebral blood flow have been shown for both indices (Li et al., 2012). The dense association between ALFF and ReHo can explain the unanimous findings in PWS patients. In addition, compared with its structural counterpart, ReHo is a more powerful biomarker for informing the underlying, regionally specific lower cerebral blood flow in PWS patients (Kochunov et al., 2025). Mantoulan et al. (2010) reported that the brain regions with hypoperfusion in individuals with PWS are mainly in the temporal lobe, cingulate gyrus, parietal lobe, and frontal lobe, agreeing with the present results indicating more extensive ReHo alterations than ALFF alterations. In summary, the use of both ALFF and ReHo may enhance the ability to detect brain regions with altered spontaneous neural activity in PWS patients. Moreover, ReHo is more sensitive than ALFF as a physiological indicator in individuals with PWS. Interestingly, several unusual findings were also revealed. The significant increases in ALFF and ReHo in the occipital and temporal lobes of pediatric PWS may be associated with the functional compensation of these brain regions. Furthermore, Prader and Willi (1956) have also reported hyperperfusion in the right orbitofrontal, bilateral middle frontal, right inferior frontal, left superior frontal, and bilateral anterior cingulate gyri of PWS patients.

### 4.2 Decreased global neural activation patterns in PWS patients

The results revealed decreased FCs between the VN and SMN, between the VN and SAN, between the SAN and DMN, and between the SMN and VN in PWS patients. The above FC results have been extensively confirmed in previous PWS research (Zhang et al., 2013; Huang et al., 2023). The VN, SMN, SAN, and DMN were designated as four of ten large-scale networks in the typical network parcellation scheme (Power et al., 2011).

The DMN has been the focus of considerable PWS research, and it has been linked to various complex cognitive processes and emotional control (Whitfield-Gabrieli et al., 2011). The DMN has higher activity levels during rest periods and lower activity levels in task states (Shulman et al., 1997). The FC showed decreased strength between the DMN and SAN, as found in previous studies (Zhang et al., 2013; Lukoshe et al., 2013). This phenomenon can be explained as the need for greater control when managing PWS patients with hyperphagia. Huang et al. (2024) reported abnormal early neurodevelopment in several brain networks, including the DMN and SAN, in children with PWS. The above may be the reason for the decrease in the FC strength between the DMN and other brain networks. In addition, a previous study (Pujol et al., 2016) reported the opposite results in adult PWS patients, which may be related to the distance effect. This phenomenon may indicate that the neurodevelopment of PWS patients changes with age. Therefore, the early detection of brain network abnormalities in patients provides an important opportunity for treatment.

The attenuated FCs between the SAN and other brain networks were predominantly in the medial cingulate and paracingulate gyri in the PWS group, which has rarely been reported before. As important parts of the SAN, the medial cingulate and paracingulate gyri are associated with negative emotions and are responsible for regulating emotional disorders (Jiang et al., 2019). Interestingly, the present results also revealed reduced functional connectivity between the medial cingulate and paracingulate gyri and the DMN, both of which are associated with emotional control. Thus, the FC changes observed within the SAN and DMN may be used as indicators for screening and regulating emotional disorders in patients with PWS.

In this study, the SMN exhibited decreased FC not only between the SMN and VN but also within the SMN itself. The SMN is also a highly valued area in PWS research. The primary function of the SMN lies in regulating and implementing motor behaviors, as well as facilitating their refinement throughout learning and developmental processes. Pujol et al. (2016) on FC in the putamen loops of the primary sensorimotor cortex in adults with PWS is linked to obsessive-compulsive symptoms like self-picking in PWS patients. PWS exhibit decreased FC in the medial VN and SMN (Huang et al., 2023), along with motor system modifications characterized by severe hypotonia in infancy (Edouard et al., 2012). Furthermore, the supplementary motor area of the SMN in PWS patients shows diminished volume as well, which may be related to central hypotonia (Ogura et al., 2011). Therefore, altered FCs in the SMNs of pediatric patients with PWS may be early physiological indicators of behavioral abnormalities in PWS patients.

The VN is primarily responsible for visual information processing and integration, ultimately forming conscious visual judgements (Cardin and Smith, 2011). The present results revealed decreased FCs between the VN and the SMN and between the VN and SAN. Visual–motor integration is significantly impaired in children with PWS (Rose et al., 2024; Lo et al., 2015), representing a side effect of abnormal alterations between the SMN and the VN. In contrast, adult PWS patients exhibit overactivation of visual brain regions when they see disgusting foods (Blanco-Hinojo et al., 2019). The subjects in the present study were too young for their visual-processing brain regions to have fully developed at the time of the study, which may have contributed to these differences. The decreased FCs within the VNs and SMNs in children with PWS may indicate small-scale intranetwork interactions (Zhang et al., 2013).

Hence, the decreased FCs in the SMNs, SANs, VNs, and DMNs of children with PWS may serve as early physiological indicators of cognitive and behavioral abnormalities, thus further reflecting underlying neurodevelopmental mechanisms.

### 4.3 Correlation analysis

The PWS brain regions that showed significant positive correlations with the GDS were located mainly in the SMN (right postcentral, right precentral gyrus, and left paracentral lobule), cingulate (left median cingulate and paracingulate gyri, right posterior cingulate gyrus, and left anterior cingulate and paracingulate gyri), and occipital lobe (bilateral calcarine). The GDS is used primarily to discriminate cognitive and motor development trends in individuals with neurodevelopmental disorders such as autism spectrum disorders (Pino et al., 2022). Previous studies have reported that the SMN, cingulate, and occipital lobes are mostly linked to symptoms, such as hypotonia, obsessive-compulsive behaviors, and emotional problems (Rice et al., 2017; Rice et al., 2016; Lieberman et al., 2019). The present study revealed that the greater the decreases in the values in the SMN, cingulate, and occipital lobes, PWS symptoms were more severe the lower the score. This phenomenon corresponds to the above PWS-related local and global brain neural activation results. The present findings suggested that alterations in the SMN, cingulate gyrus, and occipital lobe may play important roles in the underlying mechanisms leading to the emergence of emotional and behavioral problems in individuals with PWS.

### 4.4 Limitations

The present study had several limitations. First, because child templates have not been normalized and standardized relative to the widespread use of adult templates, an adult template was used in this study to avoid unstable image signals. Child-adjusted templates are the future direction of this field. Second, the age of the participants was limited to 3 months to 6 years. Further research will include more participants and longitudinal studies to provide more information on the progression of PWS.

## 5 Conclusion

The complementary and integrative fMRI approaches—ReHo, ALFF, and FC—enable comprehensive multiscale analyses to be performed. This multimodal framework facilitates the detection of subtle alterations in the activation patterns exhibited by brain regions during the early periods of PWS. In the current study, the PWS group was characterized mainly by decreased local neuronal physiological function. Both ALFF and ReHo exhibited decreases in occipital lobe, temporal lobe, and cingulate gyrus, while altered ReHo was present in parietal lobe, frontal lobe, and basal ganglia area. Decreased FC was detected in the VN, SMN, SAN, and DMN, which are designated as four of ten large-scale networks in the typical network parcellation scheme. The SMN, cingulate, and occipital lobes were significantly positively correlated with the GDS score. Thus, the present discoveries offer novel views into mechanisms of neurodevelopment associated with PWS from local neural activity and global large-scale network perspectives.

## Data Availability

The original contributions presented in the study are included in the article/Supplementary material, further inquiries can be directed to the corresponding authors.

## Footnotes

1 (RESTplus V1.32; http://restfmri.net/forum/restplus)
