# Supplementary material for: Aberrant local and global neural activation patterns in pediatric Prader–Willi syndrome
Source: Front Neurosci. 2026 Apr 15;20:1696114. doi: 10.3389/fnins.2026.1696114 (PMC13124620; doi:10.3389/fnins.2026.1696114)
Supplement: Supplementary file 1 [file Table_1.docx]

Supplementary table

| Supplementary table 1 Differences in ALFF values between PWS patients and healthy controls | | | | | |
| --- | --- | --- | --- | --- | --- |
| Brain region (AAL) | MNI coordinates | | | peak | Voxels |
|  | x | y | z |  |  |
| PWS > HC |  |  |  |  |  |
| Cluster 1 |  |  |  |  | 82 |
| Temporal_Inf_L | -51 | -45 | -9 | -5.2534 | 66 |
| Cluster 2 |  |  |  |  | 30 |
| Occipital_Mid_L | -33 | -78 | -3 | -5.7403 | 17 |
| Cluster 3 |  |  |  |  | 35 |
| Precuneus_L | -15 | -48 | 12 | -6.0461 | 12 |
| PWS < HC |  |  |  |  |  |
| Cluster 1 |  |  |  |  | 100 |
| Fusiform_R | 27 | -66 | -9 | 6.2912 | 74 |
| Lingual_R |  |  |  |  | 25 |
| Cluster 2 |  |  |  |  | 45 |
| Calcarine_L | -21 | -69 | 6 | 4.3889 | 28 |
| Lingual_L |  |  |  |  | 16 |
| Cluster 3 |  |  |  |  | 83 |
| Calcarine_R | 24 | -69 | 6 | 5.2127 | 79 |
| Cluster 4 |  |  |  |  | 155 |
| Cingulate_Mid_L | 0 | 24 | 33 | 5.6773 | 29 |
| ACC_sup_L |  |  |  |  | 60 |
| Cingulate_Mid_R |  |  |  |  | 26 |

Note: MNI, Montreal Neurological Institute; AAL, automated anatomical labeling; PWS, Prader-Willi syndrome; HC, Healthy control. *p* < 0.05, FalseDiscovery Rate (FDR) corrected, cluster > 20 voxels.

| Supplementary table 2 Differences in ReHo values between PWS patients and healthy controls | | | | | |
| --- | --- | --- | --- | --- | --- |
| Brain region (AAL) | MNI coordinates | | | Peak | Voxels |
|  | x | y | z |  |  |
| PWS > HC |  |  |  |  |  |
| Cluster 1 |  |  |  |  | 211 |
| Temporal_Inf_L | -15 | -18 | -30 | -6.2415 | 169 |
| Temporal_Mid_L |  |  |  |  | 31 |
| Cluster 2 |  |  |  |  | 86 |
| Temporal_Inf_R | 51 | -15 | -33 | -4.8614 | 73 |
| Cluster 3 |  |  |  |  | 25 |
| Occipital_Mid_L | -33 | -75 | 0 | -4.9069 | 22 |
| Cluster 4 |  |  |  |  | 43 |
| Cingulate_Post_L | -6 | -39 | 12 | -5.7826 | 13 |
| PWS < HC |  |  |  |  |  |
| Cluster 1 |  |  |  |  | 1281 |
| Calcarine_R | 24 | -66 | 6 | 6.2156 | 240 |
| Lingual_R |  |  |  |  | 283 |
| Precuneus_L |  |  |  |  | 184 |
| Precuneus_R |  |  |  |  | 166 |
| Fusiform_R |  |  |  |  | 164 |
| Cuneus_R |  |  |  |  | 54 |
| Cuneus_L |  |  |  |  | 54 |
| Cluster 2 |  |  |  |  | 256 |
| Calcarine_L | -18 | -69 | 12 | 4.658 | 106 |
| Lingual_L |  |  |  |  | 108 |
| Cluster 3 |  |  |  |  | 68 |
| Temporal_Inf_R | 48 | -66 | 0 | 3.7738 | 41 |
| Cluster 4 |  |  |  |  | 69 |
| Putamen_L | -12 | 15 | 3 | 3.5496 | 97 |
| Cluster 5 |  |  |  |  | 150 |
| Caudate_R | 15 | 3 | 18 | 4.2212 | 57 |
| Putamen_R |  |  |  |  | 75 |
| Cluster 6 |  |  |  |  | 704 |
| Cingulate_Mid_L | 0 | 21 | 36 | 6.0076 | 80 |
| ACC_sup_L |  |  |  |  | 117 |
| Cingulate_Mid_R |  |  |  |  | 115 |
| Frontal_Sup_Medial_L |  |  |  |  | 88 |
| Supp_Motor_Area_R |  |  |  |  | 79 |
| Supp_Motor_Area_L |  |  |  |  | 67 |
| Frontal_Sup_Medial_R |  |  |  |  | 41 |
| Cluster 7 |  |  |  |  | 252 |
| Frontal_Inf_Oper_R | 42 | 9 | 33 | 4.7031 | 52 |
| Frontal_Mid_R |  |  |  |  | 102 |
| Frontal_Inf_Tri_R |  |  |  |  | 61 |
| Cluster 8 |  |  |  |  | 182 |
| Frontal_Mid_L | -51 | 36 | 30 | 3.5701 | 82 |
| Frontal_Inf_Tri_L |  |  |  |  | 33 |
| Cluster 9 |  |  |  |  | 82 |
| Occipital_Mid_R | 39 | -84 | 33 | 3.8743 | 62 |
| Cluster 10 |  |  |  |  | 53 |
| Postcentral_R | 60 | -3 | 30 | 4.5349 | 46 |
| Cluster 11 |  |  |  |  | 307 |
| Precentral_R | 18 | -24 | 69 | 4.3978 | 114 |
| Postcentral_R |  |  |  |  | 52 |
| Parietal_Sup_R |  |  |  |  | 44 |
| Cluster 12 |  |  |  |  | 58 |
| Parietal_Sup_L | -36 | -54 | 66 | 4.0241 | 22 |
| Cluster 13 |  |  |  |  | 101 |
| Paracentral_Lobule_L | -3 | -24 | 63 | 3.7294 | 46 |
| Cluster 14 |  |  |  |  | 94 |
| Postcentral_L | -24 | -27 | 75 | 3.4731 | 63 |

Note: MNI, Montreal Neurological Institute; AAL, automated anatomical labeling; PWS, Prader-Willi syndrome; HC, Healthy control. *p* < 0.05, FalseDiscovery Rate corrected, cluster > 50 voxels.

| Supplementary table 3 Differences in whole brain functional connectivity based on the brain regions with differential ALFF values as seed points between PWS patients and healthy controls. | | | | | |
| --- | --- | --- | --- | --- | --- |
| Brain region (AAL) | MNI coordinates | | | Peak | Voxels |
|  | x | y | z |  |  |
| PWS < HC |  |  |  |  |  |
| ROI 1 |  |  |  |  |  |
| Calcarine_L | -21 | -69 | 6 | 4.3889 |  |
| Cluster 1 |  |  |  |  | 599 |
| Lingual_R | 6 | -75 | -9 | 6.0688 | 118 |
| Lingual_L |  |  |  |  | 135 |
| Calcarine_R |  |  |  |  | 137 |
| Calcarine_L |  |  |  |  | 120 |
| Cuneus_L |  |  |  |  | 42 |
| Cuneus_R |  |  |  |  | 27 |
| Cluster 2 |  |  |  |  | 29 |
| Temporal_Sup_L | -39 | -33 | 12 | 4.7704 | 10 |
| Rolandic_Oper_L |  |  |  |  | 13 |
| Cluster 3 |  |  |  |  | 20 |
| Temporal_Sup_R | 54 | -30 | 18 | 4.2132 | 10 |
| Cluster 4 |  |  |  |  | 33 |
| Postcentral_L | -45 | -12 | 39 | 6.5739 | 32 |
| Cluster 5 |  |  |  |  | 100 |
| Precentral_R | 18 | -24 | 66 | 4.6416 | 90 |
| Cluster 6 |  |  |  |  | 25 |
| Paracentral_Lobule_L | -6 | -24 | 63 | 5.555 | 25 |
| Cluster 7 |  |  |  |  | 22 |
| Supp_Motor_Area_R | 9 | 15 | 69 | 4.2001 | 21 |
| ROI2 |  |  |  |  |  |
| Calcarine_R | 24 | -69 | 6 | 5.2127 |  |
| Cluster 1 |  |  |  |  | 485 |
| Lingual_L | -9 | -75 | 3 | 5.9666 | 137 |
| Calcarine_L |  |  |  |  | 58 |
| Lingual_R |  |  |  |  | 79 |
| Cuneus_L |  |  |  |  | 30 |
| Cluster 2 |  |  |  |  | 46 |
| Precentral_R | 18 | -27 | 63 | 4.8074 | 45 |
| ROI 3 |  |  |  |  |  |
| Cingulate_Mid_L | 0 | 24 | 33 | 5.6773 |  |
| Cluster 1 |  |  |  |  | 181 |
| ACC_sup_L | -6 | 36 | 18 | 5.7994 | 42 |
| ACC_pre_L |  |  |  |  | 56 |
| ACC_sup_R |  |  |  |  | 33 |
| Cluster 2 |  |  |  |  | 25 |
| Lingual_L | -9 | -45 | 6 | 5.1076 | 13 |
| Cluster 3 |  |  |  |  | 43 |
| Cingulate_Post_R | -3 | -42 | 21 | 5.9836 | 22 |
| Cluster 4 |  |  |  |  | 25 |
| Cingulate_Mid_L | 0 | 21 | 36 | 4.7247 | 20 |

Note: ROI, region of interest; MNI, Montreal Neurological Institute; AAL, automated anatomical labeling; PWS, Prader-Willi syndrome; HC, Healthy control. *p* < 0.05, FalseDiscovery Rate corrected, cluster > 20 voxels.

| Supplementary table 4 Differences in whole brain functional connectivity based on the brain regions with differential ReHo values as seed points between PWS patients and healthy controls. | | | | | |
| --- | --- | --- | --- | --- | --- |
| Brain region (AAL) | MNI coordinates | | | Peak | Voxels |
|  | x | y | z |  |  |
| PWS < HC |  |  |  |  |  |
| ROI 1 |  |  |  |  |  |
| Calcarine_L | -18 | -69 | 12 | 4.658 |  |
| Cluster1 |  |  |  |  |  |
| Lingual_R | 9 | -72 | -9 | 5.7804 | 19 |
| ROI 2 |  |  |  |  |  |
| Calcarine_R | 24 | -66 | 6 | 6.2156 |  |
| Cluster 1 |  |  |  |  | 373 |
| Lingual_R | -3 | -72 | 3 | 6.0643 | 96 |
| Calcarine_L |  |  |  |  | 121 |
| Lingual_L |  |  |  |  | 65 |
| Calcarine_R |  |  |  |  | 52 |
| Cluster 2 |  |  |  |  | 30 |
| Precentral_R | 45 | -12 | 48 | 5.9042 | 28 |
| Cluster 3 |  |  |  |  | 22 |
| Postcentral_L | -45 | -12 | 39 | 5.221 | 22 |
| ROI 3 |  |  |  |  |  |
| Cingulate_Mid_L | 0 | 21 | 36 | 6.0076 |  |
| Cluster 1 |  |  |  |  | 35 |
| Cingulate_Post_R | -3 | -42 | 21 | 5.3663 | 20 |
| Cluster 2 |  |  |  |  | 257 |
| ACC_sup_L | 0 | 36 | 18 | 6.5255 | 59 |
| ACC_pre_L |  |  |  |  | 53 |
| Frontal_Sup_Medial_L |  |  |  |  | 42 |
| ACC_sup_R |  |  |  |  | 42 |
| Cluster 3 |  |  |  |  | 20 |
| Precuneus_R | 9 | -45 | 6 | 5.3547 | 12 |
| ROI4 |  |  |  |  |  |
| Paracentral_Lobule_L | -3 | -24 | 63 | 3.7294 |  |
| Cluster 1 |  |  |  |  | 76 |
| Calcarine_R | 3 | -78 | 9 | 5.0179 | 27 |
| ROI5 |  |  |  |  |  |
| Postcentral_L | -24 | -27 | 75 | 3.4731 |  |
| Cluster 1 |  |  |  |  | 20 |
| Paracentral_Lobule_L | -6 | -24 | 63 | 5.7336 | 12 |

Note: ROI, region of interest; MNI, Montreal Neurological Institute; AAL, automated anatomical labeling; PWS, Prader-Willi syndrome. *p* < 0.05, FalseDiscovery Rate corrected, cluster > 20 voxels.
